# Conversational Agents in Health Education: Protocol for a Scoping Review

**DOI:** 10.2196/31923

**Published:** 2022-04-19

**Authors:** Leigh Powell, Mohammed Zayan Nizam, Radwa Nour, Youness Zidoun, Randa Sleibi, Sreelekshmi Kaladhara Warrier, Hanan Al Suwaidi, Nabil Zary

**Affiliations:** 1 Institute for Excellence in Health Professions Education Mohammed Bin Rashid University of Medicine and Health Sciences Dubai United Arab Emirates; 2 School of Medicine Queen’s University Belfast Belfast United Kingdom; 3 College of Medicine Mohammed Bin Rashid University of Medicine and Health Sciences Dubai United Arab Emirates

**Keywords:** conversational agents, artificial intelligence chatbots, chatbots, health education, health promotion, classification, artificial intelligence assistants, conversational artificial intelligence

## Abstract

**Background:**

Conversational agents have the ability to reach people through multiple mediums, including the online space, mobile phones, and hardware devices like Alexa and Google Home. Conversational agents provide an engaging method of interaction while making information easier to access. Their emergence into areas related to public health and health education is perhaps unsurprising. While the building of conversational agents is getting more simplified with time, there are still requirements of time and effort. There is also a lack of clarity and consistent terminology regarding what constitutes a conversational agent, how these agents are developed, and the kinds of resources that are needed to develop and sustain them. This lack of clarity creates a daunting task for those seeking to build conversational agents for health education initiatives.

**Objective:**

This scoping review aims to identify literature that reports on the design and implementation of conversational agents to promote and educate the public on matters related to health. We will categorize conversational agents in health education in alignment with current classifications and terminology emerging from the marketplace. We will clearly define the variety levels of conversational agents, categorize currently existing agents within these levels, and describe the development models, tools, and resources being used to build conversational agents for health care education purposes.

**Methods:**

This scoping review will be conducted by employing the Arksey and O’Malley framework. We will also be adhering to the enhancements and updates proposed by Levac et al and Peters et al. The Preferred Reporting Items for Systematic Reviews and Meta-Analyses (PRISMA) extension for scoping reviews will guide the reporting of this scoping review. A systematic search for published and grey literature will be undertaken from the following databases: (1) PubMed, (2) PsychINFO, (3) Embase, (4) Web of Science, (5) SCOPUS, (6) CINAHL, (7) ERIC, (8) MEDLINE, and (9) Google Scholar. Data charting will be done using a structured format.

**Results:**

Initial searches of the databases retrieved 1305 results. The results will be presented in the final scoping review in a narrative and illustrative manner.

**Conclusions:**

This scoping review will report on conversational agents being used in health education today, and will include categorization of the levels of the agents and report on the kinds of tools, resources, and design and development methods used.

**International Registered Report Identifier (IRRID):**

DERR1-10.2196/31923

## Introduction

### Background

Conversational agents (CAs) are increasingly being used in various industries, including education, entertainment, and health [1]. CAs are also starting to become commonplace in formal health care settings to assist with simple functions, such as appointment scheduling and health monitoring [2,3]. As technology has progressed to enable CAs to engage in natural dialogue, we are starting to see CAs become attractive alternatives for things that have traditionally required human-to-human interaction [4]. This has enabled deployment of CAs to address more complex concerns, such as mental health support [5,6] and patient education [7,8], and in other areas in which the aim is to promote behavior change [9]. One appealing factor is the accessibility of CAs, as they enable people to access information online through a multitude of devices like computers, mobile phones, and voice-assistant hardware, such as Alexa and Siri. It is perhaps then unsurprising that CAs designed to promote health education are beginning to emerge. In the context of this paper, health education is considered education that increases awareness and seeks to favorably influence the attitudes and knowledge related to improving health on a personal or community basis [10].

### From Chatbots to CAs

The dictionary defines a chatbot as a “computer program designed to simulate conversation with human users” [11]. The first chatbot ELIZA developed by Joseph Weizenbaum in the 1960s is well established and recorded. ELIZA was an early natural language processing (NLP) chatbot, but made strides in conversational artificial intelligence (AI). It was one of the first AI programs to have passed a restricted Turing test for machine intelligence. It was broadly taken as one of the earliest successes of using intelligence in computers. ELIZA laid the groundwork for current advances in AI chatbot technology; advanced AI CAs, such as Siri and Amazon Alexa, are descendants of ELIZA [12].

Early generations of chatbots used simple pattern matching design techniques and had very basic functionality, which required specific inputs in order to generate outputs [13]. These types of chatbots were also referred to as rule-based chatbots. Later generations of chatbots saw the implementation of NLP. NLP is a subset of AI that is defined as “the ability of a computer program to understand human language as it is spoken and written (referred to as natural language)” [14]. With the introduction of NLP, simple chatbots started to shift into a generation of CAs defined by their ability to have true conversations by generating natural language in a more conversational format. The first wave of CAs emerged around 2016 when social media platforms enabled the creation of chatbots for commercial services. This led to a wave of adoption in industries ranging from health care to shopping as they often replaced the need for any human interaction [15]. Subsequent generations of CAs have progressed with advances in AI and NLP.

### Classification of CAs

As CAs have advanced, a wide diversity of terminology has been used. Terms, such as chatbot, virtual assistant, and CA, are found to be used interchangeably, leaving no clear classification method to understand what distinguishes one from another. There are many different ways to classify these initiatives, such as classification based on the depth of information CAs have access to (ie, open or closed domain) [16], the sentimental proximity of the interaction (ie, intrapersonal, interpersonal, or interagent) [1], the ways in which input is received and/or responses are generated [17], or the goal that is trying to be achieved (ie, to inform, converse, or engage the user in a task) [1]. One aim of this review is to help classify the characteristics of CAs, drawing the definitions from industry leaders like Rasa and Artificial Solutions [18-20].

For the purpose of this review, we will use a 5-level classification schema [18] adapted from characteristics detailed by Rasa, a popular open-source CA development platform [21]. The levels are as follows:

1. Level one: The CA does not have conversational ability and lacks the ability to contextualize or have a natural conversation. CAs at this level place the work on the end user. Static web forms and basic notification assistance would fall at this level.
2. Level two: The CA can conduct basic conversation through prebuilt dialogues. There is a heavy reliance on preprogramming of intents and rules. The CA can answer simple FAQs, but unexpected deviation from programmed language will lead to a lack of outcome. CAs at this level are built using rule-based dialogues and have a heavy reliance on conditional statements.
3. Level three: The CA interaction is more linguistic-based and closer to a conversation akin to an encounter between 2 humans. CAs at this level are contextual, allowing for a more flexible, almost natural back and forth conversation. CAs at this level employ NLP and natural language generation. A wide variety of chatbots today are at this level.
4. Level four: CAs at this level are sometimes referred to as consultative assistants. The onus of figuring out what the end user needs is put more on the CA instead of the end user. CAs at this level offer a more personalized experience.
5. Level five: CAs at this level are often an autonomous organization of assistants that can adjust their behavior and pick up cues.

There is no clear dichotomy between these stages, but this gradation provides us with an understanding of where CA technology stands and how it could be leveraged in health education. While scoping reviews exist about CAs in health care [21,22], this scoping review will focus on those being used within health education. The review will also provide a unique perspective in this research area by classifying interventions using emerging terms from the marketplace.

### Objectives and Review Questions

The focus for this review is on *the use of CAs in health education.* This focus was identified after a comprehensive discussion among the protocol authors, which helped clarify the key concepts that we intended to explore. Our objective to categorize the landscape of CAs in health education will be expressed in answering the following review questions:

1. What levels in the cycle are being currently used in health education CAs?
2. What are the resources needed to develop and sustain these CAs?
3. What approaches are taken in designing CAs?
4. For what health education purposes are these CAs being implemented?

## Methods

### Key Considerations

This scoping review will be guided by the methodology proposed by Arksey and O’Malley [23], with enhancements suggested by Levac et al [24] and Peters et al [25]. The Preferred Reporting Items for Systematic Reviews and Meta-Analyses Extension for Scoping Reviews (PRISMA-ScR) checklist will also provide further details and clarity [26]. As the search for a scoping review is understood to be an iterative process, any deviations from any of the predefined stages in the protocol will be noted and vindicated in the final scoping review.

### Eligibility Criteria

We will include articles that report on a CA designed and/or implemented to educate an individual or the public about an area related to health. Included papers must have details on how the CA receives input and provides output in order to enable classification. Peer-reviewed articles, conference papers, and work-in-progress papers will be included. Grey literature will also be included by conducting a google scholar search using evidence-exhaustion and effort-bounded criteria to limit the documents to be screened to the first 100 results, moving to the next hundred if the results continue to meet the eligibility criteria [27].

We will exclude studies that report on the use of CAs in nonhealth education contexts (ie, excluding medical training, medical education, continuing professional development, and educating students). Papers written in languages other than English will also be excluded.

### Search Strategy

The search strategy for this protocol was developed in a collaborative effort involving the authors and an information specialist. We aligned on a definition of “health education” by searching the MeSH (Medical Subject Headings) database, and we adopted the definition for the concept of health education that was indexed as a MeSH concept. An initial search of PubMed was conducted using our 2 main concepts of “health education” and “conversational agents.” Our list of keywords and our sample search strategy are reflected in Tables 1-3. Relevant papers were identified, and their keyword appendices were consulted to inform our list of keywords [28,29]. To ensure a thorough search strategy, we consulted with an expert in library sciences who helped to ensure our keywords are comprehensive and the select databases are relevant for this review. The databases to be searched are (1) PubMed, (2) PsychINFO, (2) Embase, (4) Web of Science, (5) SCOPUS, (6) CINAHL, (7) ERIC, (8) MEDLINE, and (9) Google Scholar.

**Table 1:** Keywords for the concept “conversational agent.”

Number (#)	Keywords
1	conversational agent*^a^
2	conversational bot*
3	conversational system*
4	conversational interface*
5	chatbot*
6	chat bot
7	chatbot*
8	chatter bot*
9	Chatterbot*
10	smart bot*
11	smartbot*
12	smart-bot*
13	virtual agent*
14	embodied agent*
15	virtual coach*
16	virtual human
17	AI bot*
18	AI-bot
19	AI Assistant*
20	Virtual Assistant*
21	Relational Agent*
22	Interactive Agent*
23	Online agent*
24	Communication agent*
25	Natural Language Generating Agent*
26	Notification Assistant*
27	FAQ Assistant*
28	Contextual Assistant*
29	Personalized Assistant*
30	Autonomous Assistant*

^a^The “*” symbol next to words helps to broaden the search through finding words that start with the same root word.

**Table 2:** Keywords for the concept “health education.”

Number (#)	Keywords
1	Patient Education*^a^
2	Health Education *
3	Health Awareness
4	Patient Awareness Programme*
5	Health Education Programme*
6	Health Advocacy*
7	Community health education*
8	Health Literacy*
9	Patient Communication*
10	Health Outreach*
11	Public Health*
12	Health Promotion*
13	mHealth
14	Mobile Health*
15	Health Teaching*
16	Health Edu*

^a^The “*” symbol next to words helps to broaden the search through finding words that start with the same root word.

**Table 3:** Sample search strategy.

Search strategy	Database	Results (n)
((“conversational agent*^a^”) OR (“conversational system*”)) OR (“conversational interface*”)) OR (“chatbot*”)) OR (“chat bot”)) OR (“chat-bot*”)) OR (“smart bot*”)) OR (“smartbot*”)) OR (“smart-bot*”)) OR (“virtual agent*”)) OR (“embodied agent*”)) OR (“virtual coach*”)) OR (“virtual human”)) OR (“AI Assistant*”)) OR (“Virtual Assistant*”)) OR (“Relational Agent*”)) OR (“Interactive Agent*”)) OR (“Communication agent*”)) OR ((“chatter”[All Fields] OR “chattering”[All Fields] OR “chatters”[All Fields]) AND “bot”[All Fields])) OR (“Chatterbot*”)) AND ((((((((((((((((“Patient Education*”) OR (“Health Education*”)) OR (“Health Awareness”)) OR (“Health Education Programme*”)) OR (“Health Advocacy*”)) OR (“Community health education*”)) OR (“Health Literacy*”)) OR (“Patient Communication*”)) OR (“Health Outreach*”)) OR (“Public Health*”)) OR (“Health Promotion*”)) OR (“mHealth”)) OR (“Mobile Health*”)) OR (“Health Teaching*”)) OR (“Health Edu*”))	PubMed	279

^a^The “*” symbol next to words helps to broaden the search through finding words that start with the same root word.

### Study Selection and Screening

After the searches in each database have been completed, all identified papers will be imported into EndNote, and duplicates will be removed using the automated features of the tool. As recommended by Levac et al [24], at least two reviewers will independently conduct a title and abstract screening to categorize what literature is eligible for a full review. Before performing a full review of the papers, papers will be uploaded to the Rayyan online platform to undergo screening. An initial review of the titles and abstracts of 5 to 10 articles will be conducted by each reviewer [30]. Results of this initial review will be compared to determine if the eligibility criteria need revision. Any changes to the criteria will be documented in the scoping review publication. After the initial review, the title/abstract of the remaining papers will be screened. Any disagreements between reviewers at this stage will be mediated and resolved with discussion and consultation with a third independent referee. Finally, papers that meet eligibility criteria will undergo a full-text review [30]. The final review will be presented in both a narrative and flow diagram format as recommended by the PRISMA ScR statement [26]. Details of the excluded studies at full-text review and vindication will be appended in the final study.

### Data Extraction and Presentation

To guide consistency in our data extraction, a template for charting characteristics, informed by the Joanna Briggs Institute (JBI) template for results extraction, has been created (Table 4). The characteristics of the chart are derived directly from the objective and review questions for the study. This template will be piloted and refined during the initial review of 5 to 10 studies conducted as part of the initial pilot.

Two reviewers will independently review and chart data for each paper. Data extraction in a scoping review is an iterative process. Therefore, this data charting form will be modified and adjusted to best meet this review’s objectives and research questions.

**Table 4:** Draft charting table.

Type of data	Details of charted data
Article information	Title, authors, date of publication, source of publication, type of study, and country of study
What health education was disseminated through the CA^a^	The data would look at chatbot-targeted health issues. For example, weight management and vaccine hesitancy.
What are the demographics of the CA end user	Age of users, country of user, and health condition (if any)
Outcomes	What outcomes does the paper mention? For example, outcomes relating to the CA or health education will be outlined here.
Resources	What resources were utilized in developing and disseminating the CA (resources including but not limited to human resources, technological resources, knowledge resources, and skill set)?
CA design	This section will extract data regarding “how.” How the CA was designed? (ie, was there a design framework used, and if so, what?) Who designed the CA? For example, health care professionals or computer scientists.
Type of CA	The CA used will be charted to 1 of 5 levels as noted previously
Duration of CA interaction	Is the chatbot used for short-term relationships, or is it only used on a one-off interaction or occasionally when a particular service is needed? Long-term relation: Is the CA providing long-term engagement? These chatbots that offer long-term engagement offer recurring updates and remember previous conversations.
Terminology	How is the CA referred to? Is it referred to as a chatbot, CA, AI^b^ chatbot, AI assistant, etc?

^a^CA: conversational agent.

^b^AI: artificial intelligence.

### Data Analysis

The analysis of the extracted data will be limited to descriptive analysis keeping it under the purview of a scoping review. We will include frequency counts of concepts and studies, which will then be mapped in various illustrations and graphs. Since this is a scoping review, the methodological quality and risk of bias will not be formally appraised or mentioned in the review.

## Results

**Figure 1:**
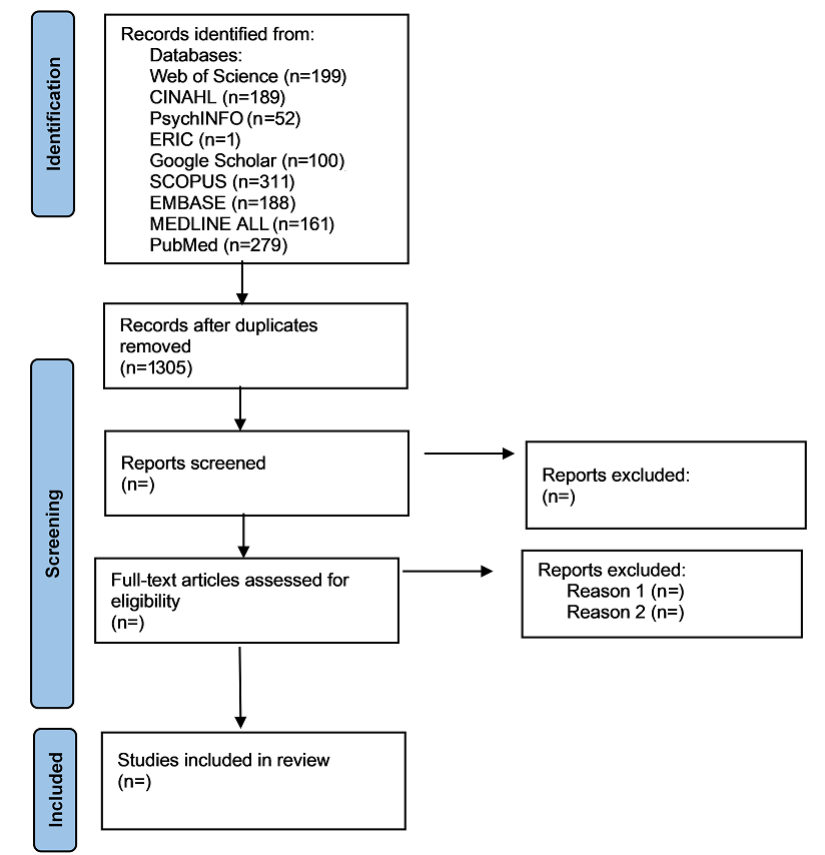
Preferred Reporting Items for Systematic Reviews and Meta-Analyses flowchart.

An initial search of the databases was conducted in September 2021, yielding 1305 results after deduplication was completed. We anticipate record screening and data extraction to be complete by mid-March 2022. The extracted results will be presented to fall in line with our review questions and objectives. Our results section will contain 2 broad sections as recommended by Peter et al [25]. The first section will include a PRISMA flow diagram that will detail the study selection process. The PRISMA flowchart is illustrated in Figure 1.

The second section will illustrate results pertaining to our review questions and objectives. A tabulation of information containing basic information of the type of study and extent of literature will be included in this section, along with a map of the extracted data. There will be a diagrammatic illustration that categorizes the types of conversations included. A narrative summary will accompany the illustrations and tables. These data presentation approaches may be further refined at the review stage.

## Discussion

Reviewers have previously explored CAs in health. However, to our knowledge, there has been no review conducted in the specific area of our interest (ie, health education). Therefore, this review will provide a map of the literature in this area, and clarify and define the heterogeneous terms found in the literature.

There are several strengths in conducting this scoping review. By categorizing existing CAs in health education into predefined categories, we seek to align on terminology that can promote more clarity in this research space. By reporting on the tools, resources, and design and development strategies undertaken to create CAs in health education, we will help to inform those seeking to develop their own CAs about what is most appropriate for their setting given their available resources, potentially leading to improved CAs and outcomes.

The limitations of this study include those inherent to scoping reviews in that we will not be formally evaluating the quality of the research. We are also limiting our scope to papers that clearly define the input/output method of the CA, which will likely result in the exclusion of papers that do not clearly discuss CA design in detail. We have determined this to be a limitation due to our aim of classifying the interventions, which is not possible with this detail. Finally, this review is limited by the fluency of the reviewers, with restriction of papers to those published in the English language.

## Abbreviations

AI: artificial intelligence
CA: conversational agent
MeSH: Medical Subject Headings
NLP: natural language processing
PRISMA-ScR: Preferred Reporting Items for Systematic Reviews and Meta-Analyses Extension for Scoping Reviews
